# Current trends in oxysterol research

**DOI:** 10.1042/BST20150255

**Published:** 2016-04-11

**Authors:** William J. Griffiths, Jonas Abdel-Khalik, Thomas Hearn, Eylan Yutuc, Alwena H. Morgan, Yuqin Wang

**Affiliations:** * College of Medicine, Grove Building, Swansea University, Singleton Park, Swansea SA2 8PP, U.K.

**Keywords:** cholestenoic acid, cholesterol, hydroxycholesterol, liver X receptor (LXR), RAR-related orphan receptor gamma t (RORγ), sterol regulatory-element binding protein (SREBP)

## Abstract

In this short review we provide a synopsis of recent developments in oxysterol research highlighting topics of current interest to the community. These include the involvement of oxysterols in neuronal development and survival, their participation in the immune system, particularly with respect to bacterial and viral infection and to T_h_17-cell development, and the role of oxysterols in breast cancer. We also discuss the value of oxysterol analysis in the diagnosis of disease.

## Introduction

Oxysterols are oxygenated derivatives of cholesterol or its sterol precursors, e.g. 7-dehydrocholesterol (7-DHC) or desmosterol [1,2]. They are formed enzymatically in the first steps of sterol metabolism and are intermediates in the formation of the steroid hormones, bile acids and 1,25-dihydroxyvitamin D_3_ [3]. Oxysterols may also be formed via non-enzymatic routes by encounters with reactive oxygen species [4,5], which provide a second pool of metabolites which also include oxidized cholesterol molecules taken from the diet [6]. A third pool may consist of oxidized cholesterol molecules generated by the gut microflora and taken up through the enterohepatic circulation. Although once thought of as inactive metabolic intermediates, the involvement of oxysterols in cholesterol homoeostasis, their role as ligands to nuclear and G protein-coupled receptors and their potential as easily measured biomarkers of disease has enhanced interest in their biosynthesis, metabolism and measurement. In this review we include in the family of oxysterols the cholestenoic acids, C_27_ carboxylated forms of cholesterol.

## Oxysterols in neuronal development survival

As the mammalian central nervous system (CNS) is rich in cholesterol and oxysterols [7], it is perhaps not surprising that oxysterols play a role in the nervous system. The most abundant oxysterol in brain is 24S-hydroxycholesterol (24S-HC), present at a level of about 20–40 ng/mg in mouse and man. This oxysterol plays a role as a cholesterol transport molecule, crossing the blood brain barrier and passing from brain to the blood stream for transport to the liver and further metabolism [8]. 24S-HC is also a ligand to the liver X receptors (LXRα and LXRβ) [9], both of which are expressed in brain, and also to the endoplasmic reticulum resident protein INSIG (insulin-induced gene) which upon ligand binding anchors the transport protein SCAP (SREBP cleavage-activating protein) along with its cargo, the pro-form of the transcription factors SREBP (sterol regulatory-element binding protein), in the endoplasmic reticulum preventing its transport to the Golgi for activation [10]. The mature, or nuclear, forms of the SREBP proteins 1c and 2 are transcription factors regulating the expression of the biosynthetic enzymes of the fatty acid and cholesterol synthesis pathways respectively [11]. It is likely that side-chain oxysterols, like 24S-HC, are important for the fine tuning of cholesterol biosynthesis, whereas cholesterol itself, through direct binding to SCAP, is more important for the coarse tuning of a negative-feedback mechanism [12,13].

**Figure 1:**
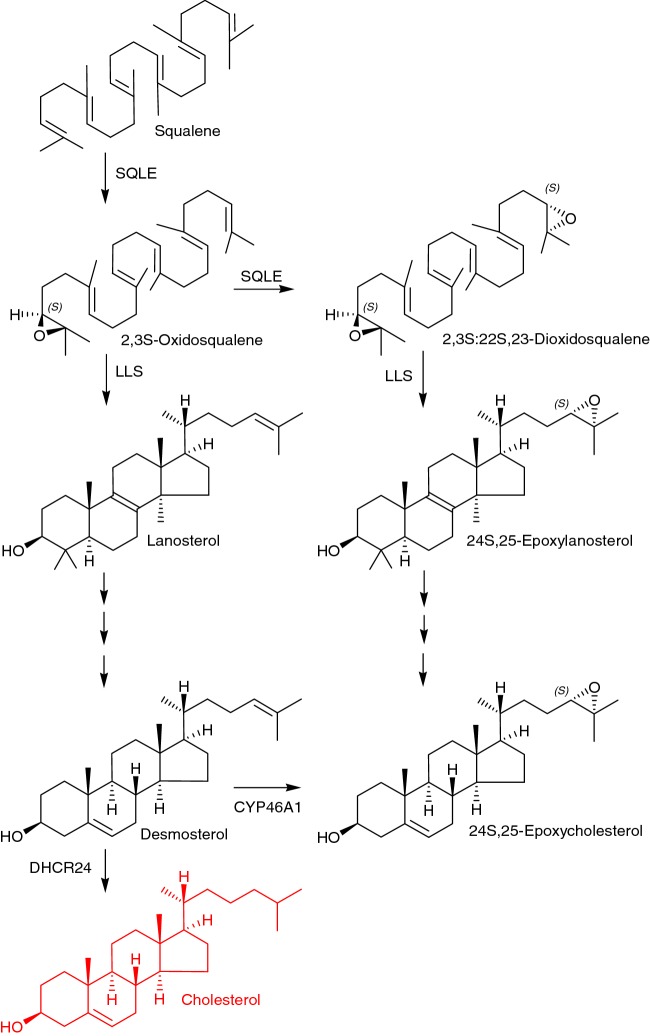
Simplified pathway from squalene to cholesterol and 24S,25-epoxycholesterol

In foetal development in mouse, cytochrome P450 (CYP) 46A1, the enzyme responsible for the metabolism of cholesterol to 24S-HC, is weakly expressed until E18 [14], and instead 24S,25-epoxycholesterol (24S,25-EC) is a dominating oxysterol (24S,25-EC, 0.3–0.4 ng/mg; cf. 24S-HC, 0.03 ng/mg at E11.5) [15]. 24S,25-EC is an unusual oxysterol in that it is synthesized via shunt pathways in parallel to cholesterol synthesis rather from cholesterol itself (Figure 1) [12]. Either, the enzyme squalene epoxidase (SQLE), also known as squalenemonooxygenase (SM), introduces one oxygen atom to squalene to give 2,3S-oxidosqualene (squalene-2,3S-epoxide) followed by cyclisation by lanosterol synthase (LLS) to lanosterol for subsequent cholesterol biosynthesis, or rather SQLE introduces a second oxygen atom to squalene to give 2,3S:22S,23-dioxidosqualene prior to cyclisation to 24S,25-epoxylanosterol, ultimately leading to 24S,25-EC. A second pathway to 24S,25-EC synthesis is from desmosterol in a CYP46A1 catalysed reaction [16]. Interestingly, it has been shown that 24S,25-EC and desmosterol, its parallel metabolite during cholesterol synthesis, are both reduced in concentration in brain from *Cyp46a1* knockout (*Cyp46a1-/-*) mice [17]. These data can be explained by either, reduced expression of enzymes of the cholesterol biosynthesis pathway in response to removal of its export route through 24S-hydroxylation and therefore enhanced negative feedback via cholesterol, SCAP and SREBP, or alternatively, and perhaps in combination, through elimination of the desmosterol to 24S,25-EC pathway catalysed by CYP46A1. Unpublished data from the authors and collaborators at Karolinska Institutet in Sweden indicate that 24S,25-EC is more abundant in transgenic mice overexpressing human CYP46A1, lending weight to the hypothesis portending synthesis via this enzyme. This pathway to 24S,25-EC synthesis may have importance in developing brain where despite low expression of CYP46A1 desmosterol levels are high [18].

24S,25-EC is both a ligand to INSIG, thus involved in regulation of cholesterol biosynthesis, and is also a potent ligand to the LXRs. Its comparative high level in developing foetal mouse midbrain (0.39 ng/mg at E11.5) points to a biological activity in this region [19]. Interestingly, midbrain progenitors cells have reduced neurogenic capacity in *LxraLxrb* double knockout mice (*Lxra-/-Lxrb-/-*), whereas overexpression of *Lxr*s promotes midbrain dopaminergic neurogenesis [20]. Recent studies have identified 24S,25-EC as a midbrain LXR ligand promoting dopaminergic neurogenesis in midbrain progenitor cells and embryonic stem cell cultures [19]. These data suggest that LXR ligands may be of value in cell replacement and regenerative therapies for Parkinson's disease, a disease in which dopaminergic neurons are lost.

**Figure 2:**
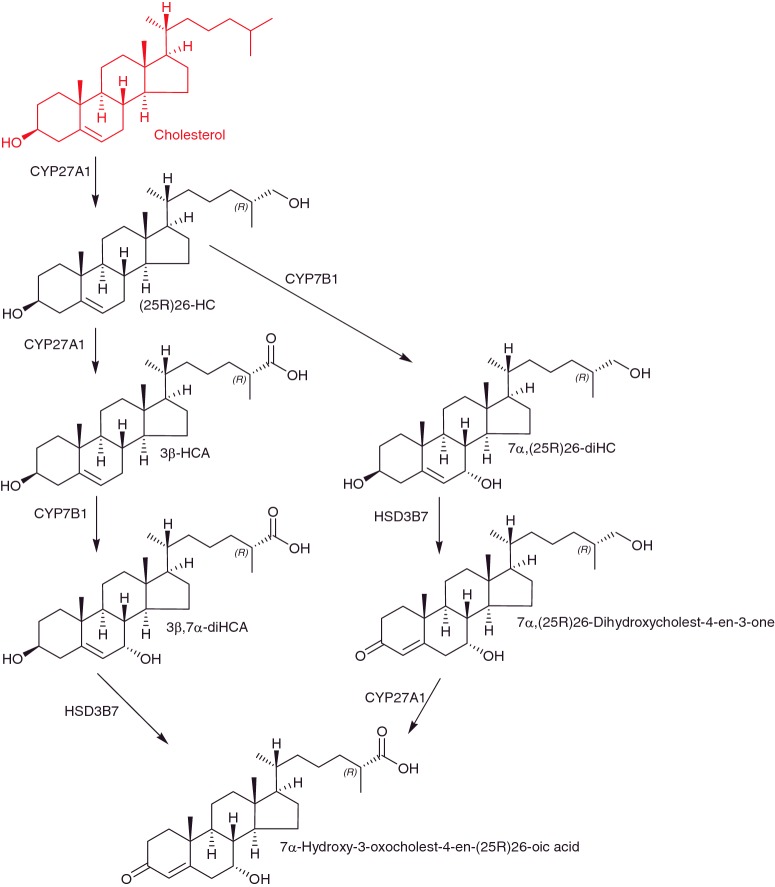
The acidic pathway of cholesterol metabolism operating in the CNS

Adult *Lxrb-/-* mice show progressive accumulation of lipids in brain and loss of spinal cord motor neurons [21], indicating that LXRs are important for survival of neurons in the adult. Besides oxysterols, cholestenoic acids are also ligands to the LXRs [22,23] and there is an expanding body of evidence indicating that cholestenoic acids are synthesized in the CNS (Figure 2). Meaney et al. [24] showed that there is a net export of 7α-hydroxy-3-oxocholest-4-en-26-oic acid from human brain to the circulation, in-part compensating for a net import of (25R)26-hydroxycholesterol ((25R)26-HC) into brain from the circulation [25]. Note, we use here systematic nomenclature where hydroxylation at the terminal side chain of cholesterol is on C-26 leading to 26-hydroxycholesterol (26-HC) which may have 25R or 25S stereochemistry [26]. Unless stated otherwise 25R stereochemistry is assumed. In much of the literature (25R)26-HC is referred to 27-hydroxycholesterol (27-HC), presumably the 25R isomer. More recently, Crick et al. [27] and Iuliano et al. [28] showed that 7α,(25R)26-dihydroxycholest-4-en-3-one, a precursor of 7α-hydroxy-3-oxocholest-4-en-(25R)26-oic acid in the pathway from (25R)26-HC is similarly exported from human brain to the circulation, and the authors group have identified low levels (0.01 ng/mg) of 3β-hydroxycholest-5-en-(25R)26-oic acid (3β-HCA) in mouse brain [29] and in collaboration with investigators at Stanford University have identified this acid and its down-stream metabolites 3β,7α-dihydroxycholest-5-en-(25R)26-oic acid (3β,7α-diHCA) and 7α-hydroxy-3-oxocholest-4-en-(25R)26-oic acid in porcine brain. All of these cholesterol metabolites can also be found in human cerebrospinal fluid (CSF) [29]. Using (25R)26-HC as a starting substrate in the pathway to 7α-hydroxy-3-oxocholest-4-en-(25R)26-oic acid the 7α-hydroxy group is introduced by the enzyme CYP7B1. Mutations in *CYP7B1* leading to a defective oxysterol 7α-hydroxylase enzyme result in the disease hereditary spastic paresis type 5 (SPG5) [30]. Patients with this disease show upper motor neuron degeneration, linking defective cholesterol metabolism to motor neuron disorder. A second cholesterol metabolic disorder, cerebrotendinous xanthomatosis (CTX) can also present with motor neuron degeneration. In CTX the (25R)26-hydroxylase enzyme, CYP27A1, is deficient, resulting in deranged cholesterol metabolism. By profiling the plasma and CSF of CTX and SPG5 patients we found that both showed a reduced level of 3β,7α-diHCA, whereas SPG5 patients showed high levels of 3β-HCA. Further *in vitro* and *in utero* studies in mouse identified 3β,7α-diHCA as a neuroprotective molecule towards motor neurons whereas 3β-HCA was neurotoxic. The neuroprotective mechanism is driven through LXR, indicating that specific cholestenoic acids selectively work on motor neurons to regulate the balance between survival and death [29].

## Oxysterols in the immune system

**Figure 3:**
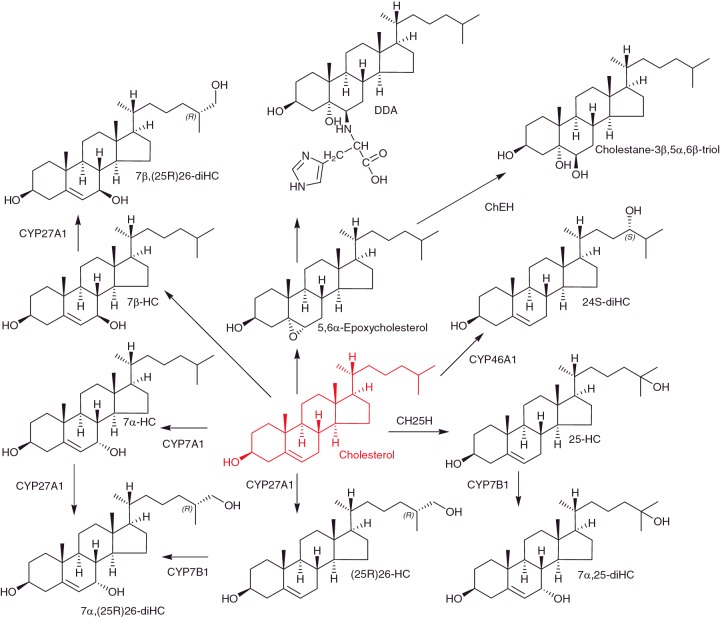
Oxysterols derived from cholesterol

25-Hydroxycholesterol (25-HC) is usually found at low levels in biological samples, and there is often doubt if it is formed enzymatically by cholesterol 25-hydroxylase (CH25H) or through *ex vivo* oxidation during sample handling and storage. However, activation of macrophages through the Toll-like receptor (TLR) by lipopolysaccharide or lipid A, mimicking bacterial infection, results in marked up-regulation of CH25H and synthesis of 25-HC both in mouse and man (Figure 3) [31,32]. Bauman et al. [31] treated naïve B-cells with nM concentrations of 25-HC and found it suppressed IL-2 mediated stimulation of B-cell proliferation, repressed activation of induced cytidine deaminase expression, and blocked class switch recombination, leading to markedly reduced IgA production. They suggested that suppression of IgA class switching in B-cells in response to TLR activation provides a mechanism for negative regulation of the adaptive immune response by the innate immune system. Blanc et al. [33] have found that 25-HC is also produced by macrophages in response to viral infection or interferon (IFN) stimulation and acts as a paracrine inhibitor of viral infection. More recently, Reboldi et al. [34] have shown that 25-HC acts as a mediator in the negative-feedback pathway of IFN signalling on IL-1 family cytokine production and inflammasome activity. *Ch25h-/-* mice were found to show increased sensitivity to septic shock, exacerbated experimental autoimmune encephalomyelitis, a mouse model for multiple sclerosis, and a stronger ability to repress bacterial growth [34]. 7α,25-Dihydroxycholesterol (7α,25-diHC) is a down-stream metabolite of 25-HC (Figure 3) and is also involved in the immune response. Hannedouche et al. [35] and Liu et al. [36] both identified 7α,25-diHC as a potent agonist of the G protein-coupled receptor EBI2 (GPR183). 7α,25-diHC was found to act as a chemoattractant for immune cells expressing EBI2 by directing cell migration. *Ch25h-/-* mice failed to position activated B-cells within the spleen to the outer follicle and showed a reduced plasma cell response after immune challenge [35].

The nuclear receptor RAR-related orphan receptor γt (RORγt) is required for generating IL-17-producing CD4^+^ T_h_17 cells which are essential in host defence and may also play pathogenic roles in autoimmune disease. CD4^+^ T-cells comprise a heterogeneous group of effector T helper (T_h_)-cells which function as the conductor, orchestrating phagocytes and B-cells to effectively clear invading pathogens. Based on their cytokine-expression profile T_h_-cells can be divided into various subtypes, including the pro-inflammatory T_h_1 and T_h_17-cells and anti-inflammatory T_reg_-cells. Multiple sclerosis, for example, is driven by an imbalance between T_h_17, T_h_1 and regulatory T_reg_-cells. Soroosh et al. [37] have identified 7β,26-dihydroxycholesterol (7β,26-diHC), presumably the 25R-epimer, as a potent agonist for RORγt. 7β,26-diHC and its isomer 7α,26-diHC both enhance the differentiation of murine and human IL-17-producing T_h_17-cells in a RORγt dependent manner [37]. Interestingly, *Cyp27a1-/-* mice, deficient in the (25R)26-hydroxylase required to generate both 7β,26-diHC and 7α,26-diHC (Figure 3) show a significant reduction in IL-17-producing cells, including CD4^+^ cells [37]. Soroosh et al. using LC–MS based technology were able to identify 7β,26-diHC and 7α,26-diHC in T_h_17-cells as metabolic products of exogenously added 7β-hydroxycholesterol (7β-HC) and 7α-HC respectively. Furthermore, *in vitro* differentiated T_h_17-cells were found to produce 7β,26-diHC [37]. These data are particularly interesting as a sterol 7β-hydroxylase enzyme has not been identified, although an alternative route may be reduction of a 7-oxo intermediate by the enzyme HSD11B1. In other studies, cholesterol precursors, rather than oxysterols, have been suggested to be RORγt ligands. Hu et al. [38] found desmosterol as a potent RORγt agonist and showed that desmosterol accumulates during T_h_17-cell differentiation as does its sulfate ester, both serving as endogenous RORγt agonists, whereas Santori et al. [39] identified cholesterol precursor(s) downstream of lanosterol but up-stream of zymosterol as RORγt ligands.

## Oxysterols as oestrogen receptor agonists

(25R)26-HC has been shown to be a selective oestrogen receptor (ER) modulator [40]. Recently, it has been shown by Nelson et al. [41] to be an ER ligand and to increase ER-dependent growth in mouse models of breast cancer. In addition, the expression of *CYP27A1* was found to correlate with tumour grade in breast cancer specimens, and in high grade tumours *CYP27A1* was expressed in tumour cells and also tumour associated macrophages [41]. CYP7B1, the enzyme which metabolizes (25R)26-HC to 7α,(25R)26-diHC (Figure 2) was found to be elevated at the mRNA level in several different human breast cancer data sets associated with better survival outcome in luminal A types [41]. Luminal A breast cancers generally express ER, so would be expected to be effected by the oestrogenic activity of (25R)26-HC. (25R)26-HC is also a ligand to the LXRs, and through this interaction was found to promote breast cancer metastasis [41]. It is not clear which other LXR ligands may have similar effects. Importantly, the study by Nelson et al. [41] links the oestrogenic and metastatic activity of (25R)26-HC with hypercholesterolaemia which is a risk factor for breast cancer in postmenopausal women. A second study by Wu et al. [42] published at about the same time also found (25R)26-HC to promote ER-positive breast cancer growth. In the study of Wu et al. (25R)26-HC was found to stimulate MCF-7 cell xenograph growth in mice, whereas in ER+ breast cancer patients the level of 26-HC was found to be higher in normal tissue than in similar tissue from controls. Furthermore, the 26-HC level was higher in tumour than healthy tissue. The increased 26-HC level in tumour tissue was explained by reduced *CYP7B1* expression [42]. Interestingly, neither 26-HC nor cholesterol levels in plasma were found to be significantly elevated in cancer patients compared with controls, but reduced expression of *CYP7B1* was associated with poorer patient survival [42]. These two studies by Nelson et al. [41] and Wu et al. [42] linking 26-HC to ERα and breast cancer are likely to stimulate detailed studies of the sterolome in breast and other cancers.

## Dendrogenin A a steroidal alkaloid

Dendrogenin A (DDA) is the product of the aminolysis reaction between 5,6α-epoxycholesterol and histamine (Figure 3) [43]. It has been found in mouse and human tissue at pg/mg levels and in plasma at ng/ml concentrations [43]. Importantly DDA is not detected in cancer cell lines, and its concentration in breast tumours is lower than controls, suggesting anti-tumour properties. DDA triggers tumour re-differentiation and inhibits tumour growth [43]. Interestingly, DDA is an inhibitor of cholesterol epoxide hydrolase (ChEH) the enzyme which hydrolyses 5,6-epoxycholesterols (5,6-EC) to cholestane-3β,5α,6β-triol [43]. ChEH is a dimer of 7-dehydrocholesterol reductase (DHCR7) and 3β-hydroxysteroid-Δ^8^-Δ^7^-isomerase (D8D7I), and acts as a high affinity binding site for the anti-tumour drug tamoxifen. Accumulation of 5,6-EC as a result of inhibition of ChEH due to tamoxifen binding is likely to contribute to tamoxifen's anti-cancer pharmacology. The discovery of DDA, a metabolite of cholesterol with anti-tumour properties, contrasts to that of (25R)26-HC, a cholesterol metabolite linked to promotion of breast cancer.

## Oxysterols as markers of disease

Unsurprisingly, plasma oxysterol profiles are markers of inborn errors of cholesterol metabolism, like CTX and SPG5, and of cholesterol biosynthesis e.g. Smith–Lemli–Opitz syndrome where DHCR7 is defective [44,45]. Perhaps more surprisingly, bile acids, down-stream metabolites, are markers of the lysosomal storage disease, Niemann–Pick type C (NPC) [46]. In 2001 Alvelius et al. [46] reported an unusual pattern of bile acids in urine from a patient with NPC. They found elevated levels of 3β-hydroxy-5-ene bile acids with a 7-oxo or 7β-hydroxy group. More recently, Porter et al. [47] reported elevated levels of 7-oxocholesterol and cholestane-3β,5α,6β-triol in plasma from NPC1 patients. This has been confirmed in numerous other studies and concentrations of cholestane-3β,5α,6β-triol have also been found to be elevated in NP type A and B patients [48]. The discovery of effective biomarkers for NPC1 is particularly significant in light of 2-hydroxypropyl-β-cyclodextrin showing promise as an intrathecal medication [49].

## Conclusions

Oxysterol research is currently gaining attention. The involvement of oxysterols in neuroscience, immunity and cancer highlights their importance in biology. Analysis of oxysterols is still challenging and care must be taken to avoid misinterpretation of data and confusion over isomer identification.

## Abbreviations

7-DHC: 7-dehydrocholesterol
(25R)26-HC: (25R)26-hydroxycholesterol
24S: 25-EC, 24S,25-epoxycholesterol
24S-HC: 24S-hydroxycholesterol
25-HC: 25-hydroxycholesterol
26-HC: 26-hydroxycholesterol
27-HC: 27-hydroxycholesterol
3β: 7α-diHCA, 3β,7α-dihydroxycholest-5-en-(25R)26-oic acid
3β-HCA: 3β-hydroxycholest-5-en-(25R)26-oic acid
5: 6-EC, 5,6-epoxycholesterol
7α: 25-diHC, 7α,25-dihydroxycholesterol
7α: 26-diHC, 7α,26-dihydroxycholesterol
7α-HC: 7α-hydroxycholesterol
7β: 26-diHC, 7β,26-dihydroxycholesterol
7β-HC: 7β-hydroxycholesterol
CH25H: cholesterol 25-hydroxylase
ChEH: cholesterol epoxide hydrolase
CNS: central nervous system
CSF: cerebrospinal fluid
CTX: cerebrotendinous xanthomatosis
CYP: cytochrome P450
DDA: dendrogenin A
DHCR7: dehydrocholesterol reductase 7
ER: oestrogen receptor
IFN: interferon
INSIG: insulin-induced gene
LXR: liver X receptor
NPC: Niemann–Pick type C
RORγt: RAR-related orphan receptor gamma t
SCAP: SREBP cleavage-activating protein
SPG5: hereditary spastic paresis type 5
SQLE: squalene epoxidase
SREBP: sterol regulatory-element binding protein
TLR: Toll-like receptor
